# Effect of soy fortification on the quality of *Mkarango* a traditional Kenyan fermented maize meal snack

**DOI:** 10.1002/fsn3.1798

**Published:** 2020-07-21

**Authors:** Prisca Linda Rapando, Charlotte Atsango Serrem, Dorcas Jepkorir Serem

**Affiliations:** ^1^ Department of Consumer Science School of Agriculture and Biotechnology University of Eldoret Eldoret Kenya

**Keywords:** children, consumer acceptability, fermentation, fortification, maize meal, soybean

## Abstract

This study aimed at improving the quality and nutrient density of a Kenyan fermented maize meal snack (*mkarango*) through soy fortification to alleviate protein‐energy malnutrition (PEM) in children. Nine variations of the snack were prepared by replacing 0, 30, and 50% maize with soy and fermenting each for 0, 3, and 5 days at ambient temperature. To establish the physico‐chemical characteristics, the proximate composition, bulk density, water, and oil absorption capacities and titratable acidity were determined. Consumer acceptability was evaluated using a panel of 55 individuals. Fortification of maize with 50% soy increased protein, lipid, and ash content by 256, 284, and 78%, respectively, while carbohydrates reduced by 30%. Fermentation increased lipids and slightly reduced carbohydrate content. Energy ranged from 1,600 to 1641 kJ/100 g. Oil absorption capacity and bulk density reduced with fortification and fermentation while water absorption capacity increased. Fermentation reduced pH by 29 and 31% after days 3 and 5, respectively. There was no significant difference at *p* ≤ .05 between liking of the fortified snacks and the conventional *mkarango*, but increase in number of fermentation days reduced the overall acceptance. All fortified snack variations meet more than half the Recommended Daily Allowance for children aged 0.5 to 10 years. Fortification and fermentation improve nutrient density of snacks in terms of proteins, lipids, and ash as well as the functional properties. Preference for all fortified snacks was above average. The soy fortified fermented snack has the potential to alleviate protein‐energy malnutrition in developing countries.

## Graphical abstract

The nutrient density and functional properties of a Kenyan fermented maize meal snack (mkarango) was improved through soy fortification to alleviate protein‐energy malnutrition (PEM) in children. Fortification and fermentation improved nutrient density in terms of protein, lipid, ash and energy as well as functional properties such as water absorption capacity, and hence, there was no difference in liking of the fortified snack by children, compared to the conventional one. All fortified snack variations meet more than half the Recommended Daily Allowance for children aged 0.5 to 10 years, and therefore, it has the potential to alleviate PEM.

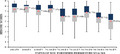

## 1 INTRODUCTION

Protein‐energy malnutrition (PEM) resulting from under nutrition is a common public health problem among poorly resourced people in developing countries. Recent global estimates show that 820 million people are food insecure and Africa has the highest prevalence of undernourishment affecting 256 million people (FAO/IFAD/UNICEF/WFP/WHO, 2019). Children are the most vulnerable to under nutrition (World Food Programme 2018). In Africa, 40% of under five‐year‐olds are stunted (Development Initiatives, 2018; FAO/IFAD/UNICEF/WFP/WHO, 2019) while Kenya has 26% stunting and 4% wasting (Kenya National Bureau of Statistics (KNBS), et al., 2015). Further, every year, PEM results in the death of about 13 million infants and children worldwide (Batool, Butt, Sultan, Saeed, & Naz, 2015), in addition to growth retardation, susceptibility to disease, poor performance, and attendance in school (Mahgoub, Nnyepi, & Bandeke, 2006). Due to poverty in the African region, many populations depend on cereals such as maize, millet, and sorghum for both their protein and energy needs because of their inability to purchase the more nutritious animal source foods (Makumba, Njobeh, Adebo, Olugbile, & Kayitesi, 2016).

Maize (*Zea mays* L) is the most important staple food crop in Eastern and Southern Africa because it accounts for almost half of proteins and kilojoules consumed (Halm, Hornbæk, Arneborg, Sefa‐Dedeh, & Jespersen, 2004). Kenya is among the highest (171g/person/day) maize consuming countries (Ranum, Pena‐Rosas, & Garcia‐Casal, 2014), and maize is a key ingredient in food products such as thin (*uji*) or thick (*Ugali*) porridges, maize, and beans (githeri), degermed maize with the testa removed (*muthokoi*) (De Groot & Kimenju, 2012) and *busaa* a traditional maize beer (Nout, 1981). It is also the most commonly purchased grain for school feeding through the Home Grown School Feeding Program (WFP, 2017). However, maize grain has poor protein quality because it is limiting in indispensable amino acids lysine and tryptophan (Ramchandran et al., 2017) required for growth and development (Shiriki, Igyor, & Gernah, 2015). Malnutrition persists in populations that heavily depend on maize (Nuss & Tanumihardjo, 2010).

Compositing cereal staples with protein‐rich legumes is a recommended means of alleviating PEM among low‐income groups (Faber, Kvalsvig, Lombard, & Benade 2005). Soybean is a legume that contains 30%–45% protein with an excellent source of all indispensable amino acids (Shiriki et al., 2015). Studies have demonstrated the improvement in protein quality of foods fortified with soybean and their positive impact on growth (Serrem, De Kock, & Taylor, 2011a; Serrem, De Kock, Oelofse, & Taylor, 2011b). Soybean flour has been used to fortify maize‐based foods with significant improvement in protein content and quality as shown by Kamau, Serrem, and Wamunga (2017). Adoption of suitable small‐scale technologies in community settings is also another suggested approach to production of low‐cost foods using locally available ingredients such as maize to meet the nutritional needs of children in developing countries (WHO, 2002). Fermentation, one of the oldest food processing and preservation technologies is indigenous to African cultures (Ross, Morgan, & Hill, 2002). Fermented foods are more nutritious, digestible, safe, improved in flavor, and shelf life (Campbell‐Platt, 1987). Additionally, modification of functional properties in aspects such as pH and reduction in bulk density are useful in preparing low bulk density foods for children (Mbata, Ikenebomeh, & Ezeibe, 2009).

*Mkarango* or *Makhalange* a traditional Kenyan fermented maize‐based snack, commonly consumed in Western Kenya (Nout, 1981: Wanjala, Onyango, Makayoto, & Onyango, 2016) is very popular among rural school children. A study by Adavachi (2017) established that the snack is also used as a weaning food and consumed in households. The snack is commonly made using 100% maize, but millet or sorghum flours can also be used. *Mkarango* is a product derived from the first stage of a two‐stage production process of *busaa,* the opaque Kenyan maize beer (Nout, 1981: Wanjala et al., 2016) or *malwa* a similar Ugandan millet beer (Muyanja, Birungi, Ahimbiswe, Semanda, & Namugumya, 2010). Stiff dough of water and raw maize or millet flour is acidified by fermenting at ambient temperatures of 22 to 30°C for 2 to 3 days. The soured dough is then roasted on a large metal sheet, stirring continuously to avoid the burnt flavor and sun dried for 2 to 3 days. Roasting partially gelatinizes the maize starch and imparts a desirable roasted flavor (Nout, 1981). The product has a long shelf life because lactic acid bacteria which colonize the snack ensure microbiological safety, while roasting and sun drying reduce its moisture content (Cheruiyot, Mbugua, Okoth, Abong, & Kaindi, 2019). Hence, the product is used as a ready‐to‐eat snack or is soaked in water before consumption (Wanjala, et al., 2016). A current advantage of *Mkarango* during the COVID‐19 quarantine period is the potential to provide recommended immuno‐supportive minerals and vitamins as a whole‐grain‐product (Muscogiuri, Barrea, Savastano, & Colao, 2020) for both children and adults in resource‐limited households.

Despite its popularity, *mkarango,* a pure maize product has low quality and nutrient density, which if enhanced by compositing with legume flour, can be more effective in meeting the protein and energy requirements of vulnerable children. Therefore, this study investigated the effect of fortifying *mkarango* with soy on nutrient, functional, and sensory characteristics, for potential use as a ready‐to‐eat snack to alleviate PEM in children.

## 2 MATERIALS AND METHODS

### 2.1 Materials

Soybean (*Glycine max*) and maize (*Zea. Mays* L) were purchased from the Eldoret Municipal market in Kenya. Sugar (Nzoia Sugar Company Ltd) and vanilla essence (Pradip East Africa Ltd) were also purchased locally.

### 2.2 Processing of maize and soybean to flours

The soybeans and maize were sorted to remove grain with damaged seed coats or which were infested by pests. Both grains were winnowed to manually separate the chaff from the grains. The soybeans were roasted for 20 min in an oven (Caterina CT/135, 5.7kw, model YXD20) at 180^0^C, while stirring occasionally. The purpose of the heating process was to reduce the levels of antinutritional factors, inactivate lipoxygenase enzymes, and improve flavor. Both the grains were then cooled at room temperature. Maize and soybean grains were later milled separately using a commercial hammer mill (Powerline®, BM‐35, Kirloskar) in Eldoret, fitted with a 2.0 mm opening screen.

### 2.3 Formulation of Snacks

Three variations of soy fortified snacks were formulated, where the first sample consisted of 100% maize (control) and the next two contained soy: maize at ratios 30:70 and 50:50, respectively. For all the samples, sugar and vanilla were added at 10% (100 g) and 2.5% (25 g), respectively. Water added depended on the treatment ranging from 11.8% (100% maize meal sample) to 21.1% (50% maize meal sample) of the total weight of ingredients based on results of preliminary experiments which established that substitution with soy meal made the slurries dry and difficult to manage requiring more water. Each variation was then subjected to different fermentation periods, 0, 3, or 5 increasing the samples to 9. Table 1 shows the 3 composites with their basic ingredients.

**Table 1:** Formulation of the soybean fortified fermented maize meal snack pastes

Ingredients	Soybean: Maize		
	0:100	30:70	50:50
Maize flour (g)	1,000 (78.4)	700 (52.8)	500 (35.1)
Soybean flour (g)	0	300 (22.6)	500 (35.1)
Water (g)	150 (11.8)	200 (15.1)	300 (21.1)
Sugar (g)	100 (7.8)	100 (7.6)	100 (7.0)
Vanilla essence (g)	25 (2.0)	25 (1.9)	25 (1.7)
Total paste weight (g)	1,275 (100)	1,325 (100)	1,425 (100)

### 2.4 Snack preparation

The dry ingredients, flour, and sugar were sieved into plastic containers and mixed with a wooden spoon for about 4 min. Water was added and mixing continued for another 3 min. Three sets of each of the three variations, soy: maize 0:100, 30:70, and 50:50 were prepared. One of each of the three variations was dry fried on a wide flat tray at medium heat of 100^0^C on an electric hotplate (Ariston DZ 02/DK 02(IX), Italy) for about 15 min with continuous stirring until light brown in color. The semi‐dried sample was then transferred to a tray into a preheated oven at 50^0^C for 20 min before sun drying until they were gritty when felt between fingers. They were then packaged in airtight zip lock plastic bags. The second and third sets of the three variations were put in airtight plastic containers and fermented for 3 and 5 days, respectively, before being dry fried, dried in the oven, and then sun dried. The samples for chemical analyses were ground using a mortar and pestle and sieved using a 1.00 mm aperture size sieve and then stored at 4^0^C until required.

### 2.5 Proximate analyses

Proximate analyses were conducted according to the standard AOAC International (1995) methods. Moisture content was determined using the oven drying procedure, method 934.1. Crude protein (N X 6.25) was determined by micro Kjeldahl, Method 992.23. Crude fat content was determined using Soxhlet extraction while ash content was determined using the dry ashing Methods 920.29 and 923.03, respectively. Carbohydrate content was calculated by difference and energy was calculated using Atwater conversion factors (FAO, 2003).

### 2.6 Functional properties

#### 2.6.1 Determination of bulk density

Bulk density was determined using the procedure described by Narayana and Rao (1984). Each of the nine samples was filled to 5 ml in a calibrated and weighed centrifuge tube by repeated tapping until there was no further change in volume. The difference in weight and volume was determined after recording the final volume and weight of sample in the tube. Bulk density was calculated as g/ml of the sample.

#### 2.6.2 Determination of water absorption capacity

Water absorption capacity was determined according to the centrifugation method of Sosulski (1962). For each sample, 2½ grams was added to a weighed 50‐ml centrifuge tube with 30 ml distilled water. The suspension was centrifuged (D72, Andreas, Hettich, Germany) at 4,000 *g* after agitating for about 5 min. The decanted supernatant was measured, including the new weight of flour and water absorbed. The difference between the new and previous weight was used to calculate the water absorption capacity and expressed as the weight of water bound by 100 g dry flour.

#### 2.6.3 Determination of oil absorption capacity

Oil absorption capacity was determined using the method of (Beuchat, 1977). One gram of each sample was added to 10 ml of pure soybean oil in a centrifuge tube, stirred for 2 min and allowed to stand at room temperature for 30 min. The samples were then centrifuged (D72, Andreas, Hettich) at 5,000 *g* for 30 min and the volume of supernatant measured in a 10‐ml graduated cylinder. The difference in volume was taken as the oil absorbed by the sample. Density of oil was taken as 0.895 g/ml.

#### 2.6.4 Determination of titratable acidity and pH

Titratable acidity was conducted according to AOAC (2000) Method 942.15. The pH of each sample was determined using a portable digital pH meter (Model HI 1,270 Checker®, Hannah Instruments) whose electrode was inserted into the suspension.

### 2.7 Consumer acceptability

Consumers who normally consume maize and soybean were recruited through an advertisement on the notice boards at the University of Eldoret, Kenya to invite 55 panelists among the staff and student population. Those who responded to the advertisement filled a consent form, where they were informed about the ingredients in the samples and were asked to ascertain their personal commitment in participating on the consumer panel to evaluate the nine variations of snacks. Only those who were not allergic to any food were allowed to participate. A random number of twenty‐four males and thirty‐one females aged between 18 and 34 years were selected (24.9 ± 6.8 years).

Each consumer was provided with all the nine variations of snacks, each served in white disposable cups on a white tray accompanied by a carrot and a glass of distilled water to cleanse their palates before and in between the tasting. The consumers were asked to rate their degree of liking for appearance, aroma, flavor color, and texture on a nine‐point Hedonic scale where 1 = dislike extremely, 5 = neither like nor dislike and 9 = like extremely. The evaluation was carried out in one session, and each session lasted 25 min. Evaluation was conducted using a completely randomized design (CRD). Each set of 9 samples was randomly assigned to the 55 panelists and the sequence of sample presentation on the tray randomized. The samples were blinded using three‐digit codes. The evaluation was done at the Department of Family and Consumer Sciences Food Laboratory at University of Eldoret where the panelists assessed the samples seated in individual stations.

### 2.8 Statistical analyses

The chemical analyses were conducted in triplicate, two different times. All data were analyzed using one‐way analysis of variance (ANOVA) and means compared using least significant difference (LSD) test with a probability of *p < *.05. Statistical analysis system (SAS) version 16 was used. The distribution of consumer Hedonic scores for the snacks was illustrated using box and whisker plots.

## 3 RESULTS AND DISCUSSION

### 3.1 Effect of soy fortification on proximate composition of fermented maize meal snack

The proximate composition of the snacks is shown in Table 2. The moisture content of the snacks ranged from 4.6% to 8.7% which is not above the recommended 10% for maize–soya blends used for supplementary feeding (WFP, 2018). The fluctuating values may be attributed to the sun drying process which varied based on the environmental conditions. Low moisture content prevents microbial activity due to low water activity, increasing shelf‐life (Omemu, Okafor, Obadina, Bankole, & Adeyeye, 2018). Additionally, removal of moisture generally increases nutrient density and availability (Amankwah, Barimah, Nuamah, Oldham, & Nnaji, 2009).

**Table 2:** Effect of soy fortification on proximate composition of fermented maize meal snacks (g/100 g) (dmb)

Maize/soy snack/ Fermentation days	Moisture	Protein (*N *× 6.25)	Crude fat	Ash	Carbohydrate^1^	Energy^2^ (kJ/g 100 g)
Maize/Soy						
Fermented (0) days						
100:0	5.6 ± 0.1^b^	6.1 ± 0.0^a^	6.9 ± 0.0^a^	1.3 ± 0.1^a^	79.9 ± 0.2^h^	1635.1^bc^
100:30	5.2 ± 0.2^b^	19.4 ± 0.2^e^	10.1 ± 0.0^d^	3.9 ± 0.1^c^	61.4 ± 0.4^d^	1638.4^bc^
50:50	4.6 ± 0.8^a^	21.7 ± 0.0^h^	12.3 ± 0.0^g^	5.0 ± 0.2^de^	56.2 ± 0.8^c^	1,660.7^d^
Fermented (3) days						
100:0	7.1 ± 0.3^d^	6.3 ± 0.0^a^	8.2 ± 0.0^b^	1.5 ± 0.1^a^	76.7 ± 0.3^g^	1633.7^b^
70:30	6.3 ± 0.1^c^	18.2 ± 0.0^c^	10.3 ± 0.0^e^	3.5 ± 0.3^bc^	61.3 ± 0.2^d^	1,630.1^b^
50:50	5.5 ± 0.0^b^	21.1 ± 0.0^f^	12.6 ± 0.0^h^	5.5 ± 0.1^e^	55.1 ± 0.1^b^	1,640.7^c^
Fermented (5) days						
100:0	8.7 ± 0.0^f^	6.8 ± 0.0^b^	9.2 ± 0.0^c^	3.1 ± 0.3^b^	65.2 ± 0.4^f^	1,600.4^a^
100:30	4.1 ± 0.0^a^	18.4 ± 0.0^d^	11.0 ± 0.0^f^	3.6 ± 0.4^bc^	62.6 ± 0.5^e^	1631.9^e^
50:50	7.8 ± 0.1^e^	21.3 ± 0.1^g^	13.6 ± 0.3^i^	4.5 ± 0.5^d^	52.6 ± 0.5^a^	1,640.8^c^

Note

Values are means ± *SD*. Values in a column followed by different letter superscripts are not significantly different at (*p* < .05) as assessed by the Least significant difference test.

^1^ Calculated by the difference method where % carbohydrates = 100‐ (fat + moisture +ash + protein).

^2^ Calcualted by multiplying with Atwater's factor where energy (kJ) = (carbohydrates × 16.736) + (protein × 16.736) + (oil × 37.656).

Fortification of maize meal with soy meal increased the crude fat content by 46.4 and 78.3% at 30 and 50% soy fortification, respectively, compared to the unfortified snack. The increase in lipid content may be explained by first, the high‐fat content of soybean flour used in this study (USDA, 2018). A similar study by Glover‐Amengor, Quansah, and Peget (2013) showed that fortification of yam flour with soybean flour also yielded a composite with 143% higher oil content than unfortified yams. The increase in lipid content may also be attributed to thermal treatment of legumes, which disrupted the lipid bodies of the soybean expelling more oil as postulated by Kayitesi, Duodu, Minnaar, and De Kock (2010) following significant increase in lipid content of sorghum flour after fortification with full‐fat marama bean.

The crude fat content in the fortified and unfortified snacks increased substantially as the fermentation days increased. Compared to the unfermented snacks fortified with soy at 0, 30, and 50%, fermentation for 3 days increased the crude fat content by 18.8, 1.9, and 2.4%, respectively, while for 5 days fermentation, the increase was 33.3, 8.9, and 10.5%, respectively. Buta and Emire (2015) also reported a 36% increase in fat content when quality protein maize meal was composited with 18% full‐fat soybean flour and fermented, for 48 hr. These researchers attributed the increase in fat content to removal of soluble carbohydrates during fermentation.

Incorporation of soy meal into the snacks dramatically increased their protein content. Replacement of maize meal flour with 30 and 50% maize meal increased the protein content by 218 and 256%, respectively, compared to the unfortified and unfermented snack. A similar trend was observed in the fermented snacks. These increases may be attributed to the high‐protein content of soybean of up to 43% (Shiriki et al., 2015). Another study by Adeyeye, Adebayo‐Oyetoro, and Omoniyi (2017) who used 5%–30% of soy protein isolate in preparation of maize flour cookies, reported similar increase of 233% protein content. Such protein increase through soybean fortification of snacks can meet the nutrient requirements of young children by half.

Substitution of maize meal with soybean flour significantly (*p* < .05) reduced the carbohydrate content of the snacks. At 30 and 50% soy substitution, carbohydrate content of the maize meal snacks reduced by 23.3 and 29.7%, respectively. The decrease can be attributed to the low carbohydrate content of soybean of up to 29% (USDA, 2018). Therefore, compositing soybean with the maize may have diluted the carbohydrate content, similar to the study by Serrem et al. (2011a) on soy–sorghum biscuits and Kamau (2015) for soy fortified complementary porridges made from millet, maize, sorghum, cassava, and banana.

The energy content of the soybean fortified snacks ranged from 1600–1641 kJ/100 g, which is almost four times the recommended minimum quantity of 400 kcal for supplementary feeding for young children (FAO/WHO, 1994). This is explained by the increase in fat content, which provides higher energy density of 37 Kj/g and also the decrease in the carbohydrate content of the snacks. High dietary energy is important for sparing protein for body building and repairing body tissues avoiding diversion to provide energy (Michaelsen et al., 2009).

Fortification with soybean flour significantly increased the ash content by 200% and 285% for the unfermented samples at 30% and 50% levels of fortification, respectively. The increase in ash content is due to the higher mineral content in soybean than maize (USDA, 2018). Serrem et al. (2011a) who replaced 28.6 to 71.4% sorghum flour with defatted soy flour in biscuits reported increased ash (mineral) content of 50 to 136% compared to the 100% cereal biscuits. Soybean flour contains high amounts of potassium, moderate levels of calcium, phosphorus and magnesium and traces of selenium, iron, zinc, and sodium, necessary micronutrients for children's health (Thompson, Manore, & Vaughan, 2011).

### 3.2 Percent contribution of Energy and Protein content of Soy fortified fermented maize meal snack per 100 g toward RDA of children aged 0.5 to 10 years

Table 3 shows the contribution of the soy fortified fermented maize meal snack to the required daily allowance (RDA) of protein and energy for children aged 0.5 to 10 years. Results show that 100 g of the unfortified snack (*Mkarango*) whether fermented for 3 or 5 days or unfermented did not meet half the protein RDA for children of all ages with the highest at 48.5%. Additionally, the contribution reduced to less than quarter (21.8%–24.5%) of the RDA as the children's age increased to 10 years. The energy contribution also reduced with age from 45%–46.7% at 0.5 to 1 year to 19.1%–19.8% at 7 to 10 years. The reduction in energy and protein is due to increased requirements for metabolism, growth, and development (Thompson et al., 2011). Compositing maize: soy at 70:30 and 50:50 dramatically increased the snacks contribution ranging from 14% to 55%, respectively, above the RDA of protein for children aged 0.5 to 3 years. For children aged 4 to 10 years, more than half 65 to 90% of RDA can be met by the fortified snacks. The contribution of protein above the RDA for 0.5‐ to 3‐year‐olds is within tolerable limits and not toxic as the recommended intake should not be more than twice the RDA (Food & Nutrition Board, 1989). From these findings, the fortified *mkarango* more than adequately meets half of the protein intake for children aged 0.5 to 10 years.

**Table 3:** Percent Contribution of energy and protein content from 100 g of soy fortified fermented maize snack to RDA for children aged 0.5–10 years

Nutrient	Age group (years)	RDA^1^	Percent contribution of soy fortified fermented maize snack (*mkarango*) to RDA								
			Fermented (0) days			Fermented (3) days			Fermented (5) days		
			% Level of fortification with soybean flour								
			0	30	50	0	30	50	0	30	50
Energy/kJ/day	0.5–1	3,556.4 (850)	46.0	46.1	46.7	45.9	45.8	46.1	45.0	45.8	46.1
	1–3	5,439.2 (1,300)	30.1	30.1	30.5	30.0	30.0	30.2	29.4	30.0	30.2
	4–6	7,531.2 (1,800)	21.7	21.8	22.1	21.7	21.6	21.8	21.3	21.7	21.9
	7–10	8,368 (2000)	19.5	19.6	19.8	19.5	19.5	19.6	19.1	19.5	19.6
Protein g/day	0.5–1	14	43.5	138.0	155.0	45.0	130.0	150.7	48.5	131.4	152.9
	1–3	16	38.1	121.3	135.0	39.3	113.8	131.9	42.5	115.0	133.1
	4–6	24	25.4	80.8	90.4	26.3	75.8	87.9	28.3	76.7	88.8
	7–10	28	21.8	69.3	77.5	22.5	65.0	75.3	24.3	65.7	76.1

Note

Samples are fortified fermented maize snacks with 0, 30, and 50% soy replacement, fermented for 0, 3, and 5 days.

^1^ Food and Nutrition Board (1989), figures in parentheses are energy/kcal/day.

### 3.3 Effect of soy fortification on functional properties of fermented maize meal snack

Results of the functional properties for the snacks are shown in Table 4. There was a marked increase in titratable acidity and decrease in pH of snack samples with increase in fermentation days and amount of soybean. For example, fortification of 100% maize meal snack with 50% soy increased titratable acidity and reduced pH by 300 and 15%, respectively, while fermentation for 3 days increased titratable acidity by 100% and reduced pH by 46.3%. This may be explained by hydrolysis of carbohydrates followed by increase in concentration of fatty acids, phosphoric acids, hydrogen ions (H^+^), and the carboxyl groups of protein amino acids following the fermentation process (Tchikoua, Tatsadjieu, & Mbofung, 2015). Reduction in pH due to fortification could also be attributed to the buffering effect of proteins as a result of the higher content of amino acids contributed by the soybeans as hypothesized by Plahar, Nti, and Annan (1997). Metabolites from Lactic acid bacteria, which increase titratable acidity and reduce pH, are inhibitory to other microorganisms (Adams, 1990; Powell, 2006) and hence increase shelf life and improve microbiological safety of lactic acid fermented foods, such as *mkarango*.

**Table 4:** Effect of soy fortification on functional properties of fermented maize meal snack (dmb)

Maize/soy snack/Fermentation days	pH	Titratable acidity	Bulk density (g/ml)	Water absorption capacity (g/100 g)	Oil absorption Capacity (g/ml)
Maize/Soy					
Fermented (0) days					
100:0	6.0^i^	0.1^a^	0.8^h^	137.2^a^	1.7^g^
70:30	5.8^g^	0.2^b^	0.7^f^	140.3^b^	1.5^g^
50:50	5.2^h^	0.4^e^	0.6^c^	142.4^d^	1.2^d^
Fermented (3) days					
100:0	4.1^b^	0.2^b^	0.7^g^	140.3^b^	1.7^g^
**70**:30	4.0^f^	0.41^d^	0.69^d^	142.3^c^	1.4^e^
50:50	4.0^f^	0.78^g^.	0.57^b^	145.4^f^	1.1^c^
Fermented (5) days					
100:0	4.0^a^	0.3^c^	0.70^e^	142.4^d^	1.3^i^
100:30	4.4^c^	0.59^f^	0.64^c^	144.4^e^	1.1^b^
50:50	4.5^d^	0.86^h^	0.52^a^	146.4^g^	0.9^a^

Note

Values are means. Values in a column followed by different letter superscripts are significantly different at (*p* < .05) as assessed by the least significant difference test.

Fermentation decreased bulk density by 13 and 25% for samples fermented for 3 and 5 days, respectively. The reduction is possibly a reflection of the action of alpha‐amylase enzyme activated during the fermentation process and dextrinization of starch to its constituent subunits, resulting in reduced fiber content (Chelule, Mokoena, & Gqaleni, 2010). Fortification of samples with soy meal at 30 and 50% decreased bulk density by 14 and 29%, respectively, after 3 days fermentation and 20 and 35%, respectively, after 5 days. The unfortified maize grain had the highest bulk density. This may be due to the whole‐grain maize used in this study, hence, the high fiber content (USDA, 2018). Low bulk density in preparation of foods for children enhances nutrient and calorie density per feed (Akpata & Akubor, 1999; Mbata et al., 2009); therefore, the decreased bulk density of the snack is an advantage.

The water absorption capacity increased significantly at *p* < .05 with increase in soy fortification and fermentation days. Proteins have hydrophilic parts such as polar or charged side chains (Adebowale & Lawal, 2004) accounting for the increased water absorption capacity as soy level increased in snacks. Increase in the fermentation days possibly led to increase in the number of microorganisms with proteolytic activity resulting in increased availability of protein functional groups in the flour, which may have increased the availability of polar groups in proteins, also increasing the hydrophilicity of flour proteins (Ohizua et al., 2017). Water absorption capacity indicates the degree to which water can be added to a food and also gives an indication of the amount of water available for gelatinization (Ohizua et al., 2017). This is important in this study as the snack requires hydration before consumption by the children.

Fortification significantly reduced the oil absorption capacity by 25% for 30% fortification and 27% for 50% fortification. The decrease may be attributed to the high oil content in soy meal (USDA, 2018) which increased as the soy increased, saturating the sample. The oil holding capacity is desirable for foods meant to be used for supplementary feeding because oil is energy dense providing 37Kj/100 g (FAO, 2003).

### 3.4 Effect of soy fortification on Consumer perception of snacks

Table 5 shows consumer perception of the sensory attributes of appearance, smell, flavor, and texture for the soy fortified and fermented maize meal snacks. The 100% maize snack fermented for 3 days, and the unfermented snacks with 0 and 30% soybean scored highest for appearance, while the 50:50 soy: maize snack fermented for 5 days was the least liked. The low score was probably a result of consumers’ unfamiliarity with the intense brown color due to the increased percentage of soybean flour. The traditional *mkarango* is light brown. A study by Ng'ong'ola‐Manani, Mwangwela, Schüller, Østlie, and Wicklund (2014) also established that brown color in a soy fortified maize‐based fermented snack was one of the major drivers for dislike by consumers. Similarly, Otegbayo, Adebiyi, Bolaji, and Olunlade (2018) reported decreased general acceptability of soy enriched bread with increased level of soybean substitution.

**Table 5:** Effect of soy fortification of fermented maize meal snack on consumer perception of sensory attributes

Maize/soy snack/ Fermentation day	Appearance	Smell	Flavor	Texture	Rank
Fermented (0) days					
Maize/Soy					
100:0	7.6 ± 1.6^cd^	7.1 ± 1.5^ef^	7.7 ± 1.7^e^	7.1 ± 1.7^bc^	6.5 ± 2.6^c^
70:30	7.5 ± 1.5^cd^	7.3 ± 1.6^f^	7.5 ± 1.6^e^	7.0 ± 1.6^bc^	5.9 ± 2.3^c^
50:50	7.1 ± 1.5^b^	6.6 ± 1.5^cde^	6.5 ± 1.7^cd^	6.7 ± 1.9^bc^	5.6 ± 1.9^c^
Fermented (3) days					
100:0	8.0 ± 1.0^d^	7.3 ± 1.4^f^	7.5 ± 1.3^e^	7.4 ± 1.4^c^	6.3 ± 2.3^c^
100:30	6.3 ± 1.7^b^	6.3 ± 1.6^bcd^	5.9 ± 2.2^bc^	7.4 ± 1.4^c^	4.1 ± 2.0^b^
50:50	5.9 ± 1.7^b^	6.0 ± 2.0^abc^	5.6 ± 2.0	5.6 ± 1.9^ab^	4.1 ± 2.6^b^
Fermented (5) days					
100:0	7.3 ± 1.3^c^	6.7 ± 1.6^ef^	6.9 ± 2.0^de^	7.0 ± 1.6^bc^	5.8 ± 2.3^c^
100:30	6.3 ± 1.8^b^	5.8 ± 2.1^ab^	5.8 ± 2.5^bc^	5.7 ± 2.2^abc^	3.9 ± 2.2^b^
50:50	5.1 ± 2.3^a^	5.5 ± 2.1^a^	4.6 ± 2.5^a^	4.9 ± 2.3^a^	2.9 ± 2.2^a^

Note

Values are mean ± *SD*. Values followed by different letter superscripts in a column are significantly different at *p* ≤ .05 as assessed by Fisher's least significant test. 9 = Like extremely, 8 = Like very much, 7 = Like moderately, 6 = Like slightly, 5 = Neither like nor dislike, 4 = Dislike slightly, 3 = Dislike moderately, 2 = Dislike very much, 1 = Dislike extremely. Consumers *n* = 55.

Consumer preference for the corresponding aroma/smell and flavor decreased with increase in soy fortification and days for fermentation of the snack. Hence, the best liked smell and flavor were for 100% maize and 30:70 soy: maize, both unfermented or fermented, while the least liked snack developed was with 50% replacement of maize with soy and fermented for 5 days. The unfermented snacks did not produce volatile compounds (Kobayashi, Tsuda, Hirata, Kubota, & Kitamura, 1995) like fermented samples and were therefore less acidic. Also, roasting probably released flavor compounds from Maillard and caramelization reactions during frying and baking (Bastos, Monaro, Siguemoto, & Séfora, 2012) of the maize–soy–sugar composite enhancing acceptability.

**Figure 1:**
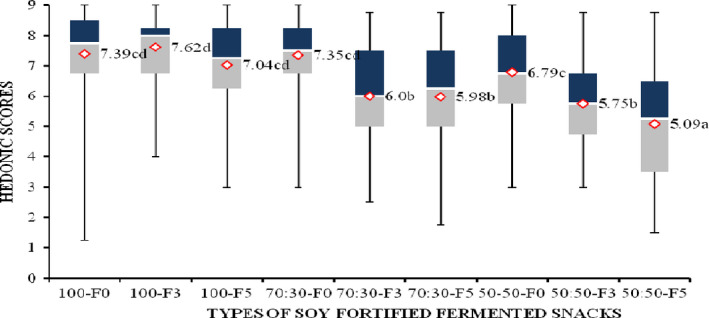
Total quality of the soybean fortified fermented maize meal snacks. ^a, b, c, d^ demonstrate that values with different letter superscripts differ significantly at *p* < .05 as assessed by Fisher's least significant test. The dark shaded area is the higher percentile and represents the value above which 75% of the ratings fell. The light shaded area is the lower percentile and represents the area where 25% of the ratings fell. The median is the thin line between the two shaded areas where 50% of the values fell above and 50% below. Overall liking ratings 1 = Dislike extremely, 2 = Dislike very much, 3 = Dislike moderately, 4 = Dislike slightly, 5 = Neither like nor dislike, 6 = Like slightly, 7 = Like moderately, 8 = Like very much, 9 = Like extremely. Consumers *n* = 60

The low score for snack with maize: soy at 50%: 50% fermented for 5 days, may have been due to the sourness and bitter aftertaste associated with fermented foods. Use of full‐fat soybean flour such as that used in this study produces high acid content (Griffith, Castell‐Perez, & Griffith, 1998). Soy flour addition beyond 30% decreases liking (Sikuku, 2017). Liking of the texture for the maize: soy 50%: 50% snack fermented 5 days was the least while all the other snacks did not differ in liking. This could be attributed to the high soy and acid content which reduced the liking as stated earlier. Results of total quality in Figure 1 show that this sample had the lowest total quality and was significantly different from the rest of the samples. All the samples with soy, which were subjected to fermentation were less liked than the samples, which had no soy and were fermented. However, the liking of all the samples was above average, an indicator that they could be acceptable to consumers.

## 4 CONCLUSION

Fortification with soybean markedly increases the nutrient density in terms of protein, lipid, and ash (mineral) content. Fortification with soybean and fermentation produce positive functional properties related to bulk density, water absorption capacity, and acidity. Soybean fortified snacks are liked as much as the conventional *mkarango*. Fortified fermented snacks have considerable potential for use as ready‐to‐use supplementary foods for alleviating PEM among young children in Kenya and other developing countries.

## CONFLICT OF INTEREST

The authors declare that they do not have any conflict of interest.

## ETHICAL APPROVAL

Approval to conduct this study was granted by the National Commission for Science, Technology and Innovation (NACOSTI) of Kenya (Permit Number: NACOSTI/P/17/27159/15643).

## INFORMED CONSENT

An informed and written consent was sought from the participants before the evaluation commenced.
